# Effects of Buffer Gases on Graphene Flakes Synthesis in Thermal Plasma Process at Atmospheric Pressure

**DOI:** 10.3390/nano10020309

**Published:** 2020-02-11

**Authors:** Cheng Wang, Ming Song, Xianhui Chen, Dongning Li, Weiluo Xia, Weidong Xia

**Affiliations:** 1 Department of Thermal Science and Energy Engineering, University of Science and Technology of China, Hefei 230027, China; awcheng@mail.ustc.edu.cn (C.W.); chenxian@mail.ustc.edu.cn (X.C.); ldn1900@mail.ustc.edu (D.L.); 2 Department of Materials Science and Engineering, University of Science and Technology of China, Hefei 230027, China; mings@mail.ustc.edu.cn; 3 Hefei Institutes of Physical Science, Chinese Academy of Sciences, Hefei 230031, China; xiawl@ipp.ac.cn

**Keywords:** graphene flakes, thermal plasma, magnetically rotating arc plasma, buffer gas, nitrogen-doped graphene flakes

## Abstract

A thermal plasma process at atmospheric pressure is an attractive method for continuous synthesis of graphene flakes. In this paper, a magnetically rotating arc plasma system is employed to investigate the effects of buffer gases on graphene flakes synthesis in a thermal plasma process. Carbon nanomaterials are prepared in Ar, He, Ar-H_2_, and Ar-N_2_ via propane decomposition, and the product characterization is performed by transmission electron microscopy (TEM), Raman spectroscopy, X-ray diffraction (XRD), X-ray photoelectron spectroscopy (XPS), and the Brunauer–Emmett–Teller (BET) method. Results show that spherical particles, semi-graphitic particles, and graphene flakes coexist in products under an Ar atmosphere. Under an He atmosphere, all products are graphene flakes. Graphene flakes with fewer layers, higher crystallinity, and a larger BET surface area are prepared in Ar-H_2_ and Ar-N_2_. Preliminary analysis reveals that a high-energy environment and abundant H atoms can suppress the formation of curved or closed structures, which leads to the production of graphene flakes with high crystallinity. Furthermore, nitrogen-doped graphene flakes with 1–4 layers are successfully synthesized with the addition of N_2_, which indicates the thermal plasma process also has great potential for the synthesis of nitrogen-doped graphene flakes due to its continuous manner, cheap raw materials, and adjustable nitrogen-doped content.

## 1. Introduction

Graphene is a novel nanomaterial with a single layer of carbon atoms packed in a hexagonal lattice. Since the first synthesis in 2004, graphene has emerged as highly active research field due to its fascinating physical, chemical, and mechanical properties [1,2,3,4]. In the past decade, various graphene preparation methods were developed such as mechanical cleavage [1], chemical vapor deposition [5,6], epitaxial growth [7,8], oxidation reduction [9], and arc-discharge method [10], among others. However, these methods generally operate in a batch mode. It remains challenging to obtain high-quality graphene economically, continuously, and with high throughput [11,12,13].

In recent years, a thermal plasma process at atmospheric pressure was developed for continuous synthesis of graphene flakes [14]. In this synthesis process, a carbon-containing precursor is delivered directly into the thermal plasma region where the precursor is decomposed to smaller reactive fragments. Then these reactive fragments recombine in the plasma environment to form graphene flakes and other products. Compared with the traditional graphene preparation methods such as chemical vapor deposition (CVD) and the arc-discharge method, this process is a single-step, rapid, continuous method that occurs at atmospheric pressure without the use of substrates, catalysts, solvents, or acids. Thus, it is considered a promising process for graphene flakes synthesis. The effects of process parameters on the graphene flakes synthesis in a thermal plasma process have been reported in some studies. For example, Dato et al. [14,15,16,17], Tatarova et al. [18,19,20], and Melero et al. [21,22] established a single-step method to synthesize graphene flakes with few layers based on microwave plasma. Their research revealed that factors influencing graphene flakes synthesis include the precursor type, reactor design, flow rate of buffer gas, etc. Pristavita et al. [23,24,25,26,27,28] and Cheng et al. [29] developed a radio-frequency plasma system for graphene flakes synthesis via methane decomposition. Their study indicated that a high reaction temperature and addition of H_2_ may promote the transformation of products from spherical particles to graphene flakes. Kim et al. [11] and Suh et al. [12,30] proposed an atmospheric arc plasma process for graphene flake preparation. Relevant research has also uncovered the effects of reaction temperature and precursors on graphene flake formation. Recently, we developed a magnetically rotating arc plasma system for continuous synthesis of graphene flakes [31,32,33]. The high yield and low energy cost make this method competitive among these thermal plasma processes. Moreover, the influence of the magnetic field, gas temperature, precursor type, and precursor flow rate on the product microstructure is systematically presented. In reviewing these studies, it is found that process parameters such as precursor composition, reaction temperature, and buffer gas are essential for preparing graphene flakes in a controlled manner. However, to the authors’ knowledge, few reports have systematically addressed the dependence of different buffer gases on the graphene flakes synthesis in the thermal plasma process even though the effects of buffer gases have been widely reported in terms of the arc-discharge method [34,35,36,37,38,39,40] and chemical vapor deposition [41,42,43].

In this paper, a magnetically rotating arc plasma system is applied to study the effects of buffer gases on graphene flakes synthesis in the thermal plasma process. Carbon nanomaterials are synthesized under different buffer gases (i.e., Ar, He, Ar-H_2_, and Ar-N_2_) via propane decomposition. Transmission electron microscopy (TEM), Raman spectroscopy, X-ray diffraction (XRD), X-ray photoelectron spectroscopy (XPS), and the Brunauer–Emmett–Teller (BET) method are employed to analyze the microstructure of the products. Based on product characterization, the influence mechanism of buffer gases on the graphene flakes formation is discussed.

## 2. Experimental Methods

### 2.1. Experimental Apparatus

**Figure 1:**
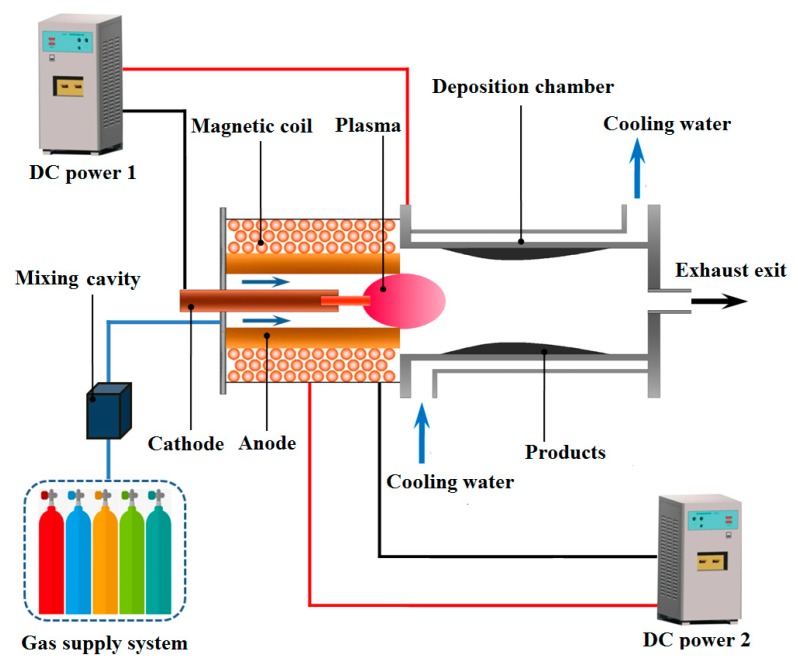
Schematic diagram of the experimental apparatus.

A schematic diagram of the experimental apparatus is exhibited in Figure 1, which is mainly composed of a magnetically rotating arc plasma generator, a water-cooling deposition chamber, a gas supply system, and a direct current (DC) power system. The plasma generator is constructed with two concentric graphite electrodes (Cathode: 8 mm in diameter and 250 mm in length. Anode: 30 mm in inner diameter and 300 mm in length) and a water-cooling magnetic coil that surrounds the anode. Buffer gas and feedstock gas are introduced into the plasma generator from gas inlets around the cathode bottom. Two modulated 0–200 V DC power supplies are connected to the plasma generator and magnetic coil, respectively. The magnetic coil provides an axial magnetic field of 0.08 T by controlling the coil current. A water-cooling deposition chamber (stainless steel, cylindrical structure, 40 mm in inner diameter, and 400 mm in length) is connected to the anode. An exit is open to the atmosphere for the exhaust emission. Thus, the deposition pressure is approximately 1 atm. A more detailed description of the experimental apparatus can be found in the authors’ previous work [31,44,45,46].

### 2.2. Experimental Parameters

The input conditions used for each test are listed in Table 1. The buffer gases include argon, helium, a mixture of argon and hydrogen, and a mixture of argon and nitrogen, respectively. The feedstock gas is propane, and the purity of all gases is more than 99.99%. To minimize the effect from a gas temperature difference, the average gas temperature is controlled as the same for different buffer gases by adjusting the input power. The average gas temperature is calculated by the energy equilibrium (assuming the system is in a thermodynamic equilibrium and the propane is adequately mixed up with buffer gas. The thermal efficiency of the plasma generator ranges from 40–60%). As suggested by Pristavita et al. [23,27] and Wang et al. [31], the essential gas temperature for graphene flakes synthesis is more than 3000 K. Therefore, the average gas temperature in this experiment is controlled to be 3500 K, and the temperature error is roughly ± 300 K. The input power is controlled by changing the arc current.

During the course of the experiment, the argon is first injected into the plasma generator, and the arc is ignited under a pure Ar atmosphere. Then the argon is replaced by the buffer gas. Lastly, the propane is mixed with the buffer gas in the mixing cavity and then introduced into the plasma generator for decomposition. The typical duration time of each test is 20 min. A mass of solid products are deposited on the inner wall of the deposition chamber. The solid products are powdery and pile together loosely. The packing density of resultant solid products is 0.1–0.12 g/cm^−3^, and the synthesis rate is about 100–300 mg per minute. The solid products are continuously prepared in a gas phase, so the formation time from feedstock gas to solid products is about 20 ms (i.e., residence time of feedstock gas in the high temperature region).

### 2.3. Characterization

The product morphology is characterized by transmission electron microscopy (TEM, JEM-2011, JEOL, Tokyo, Japan) and high-resolution transmission electron microscopy (HRTEM, JEM-2100F, JEOL, Tokyo, Japan). The crystalline structure of the products is analyzed using an X-ray diffractometer (TTRIII, Rigaku, Tokyo, Japan) and a Raman spectrometer (LabRam HR Evolution, Horiba Scientific, Villeneuve d’Ascq, France). Raman spectrum measurements are carried out by the 532-nm line of an He-Ne laser as the excitation source in the spectral range of 500–3000 cm^−1^. X-ray diffraction (XRD) measurements are performed using a Cu-K*α* radiation in the 2θ range of 10 to 70°. The elemental analysis of the products is conducted using X-ray photoelectron spectroscopy (XPS, Axis Ultra DLD, Kratos, Manchester, England). XPS spectra are obtained via a monochromatic Al irradiation source in the range of 200–700 eV. Nitrogen adsorption–desorption isotherm measurements of the products are performed using a surface area analyzer (TristarII3020M, Micromeritics, Atlanta, USA). The Brunauer–Emmett–Teller (BET) method is used to calculate the specific surface area of the products. 

## 3. Results

### 3.1. TEM Images

**Figure 2:**
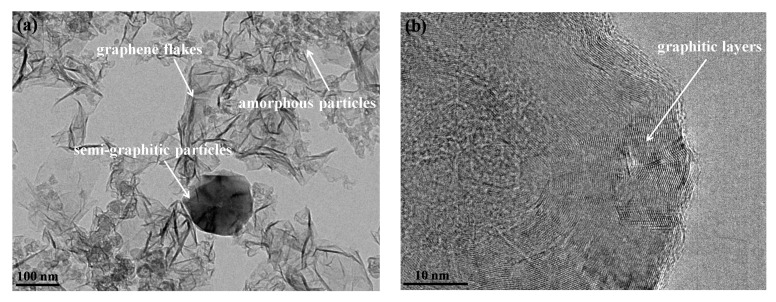

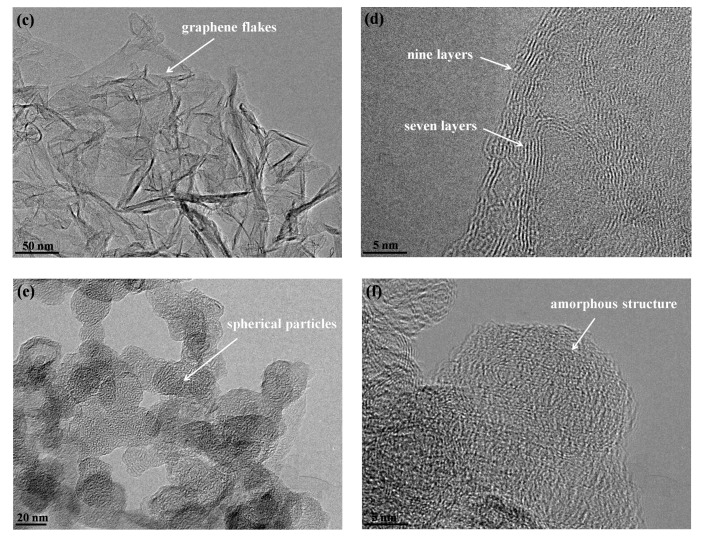
TEM images of products obtained under an Ar atmosphere. (**a**) Low-magnification TEM image of whole products. (**b**) HRTEM image of semi-graphitic particles. (**c**,**d**) TEM image of graphene flakes. (**e**,**f**) TEM image of spherical particles.

Typical TEM images of the products obtained under an Ar atmosphere are shown in Figure 2. Figure 2a indicates the existence of three kinds of products: semi-graphitic particles, graphene flakes, and spherical particles. The semi-graphitic particles have a characteristic of graphitized forms of carbon blacks [47]. These particles are stacked by dozens of graphitic layers that define the particle boundary, which leads to a polyhedral morphology and shell-like appearance, as shown in Figure 2b. This is also known as polyhedral graphite [48] or semi-graphitic polyhedral particles [49]. The size of semi-graphitic particles is often within the range of 50–150 nm, and the number of graphitic layers can range from 50 to several hundreds. The graphene flakes are the main products under an Ar atmosphere. These flakes have the size (length and width) in the range of 50–200 nm, and appear as irregularly-curled flakes that overlap and aggregate, which is, hence, being labeled “crumpled paper sheet”-like carbon black [50,51,52,53], as revealed in Figure 2c,d. TEM image analysis indicates that the graphene flakes consist of graphitic layers with the number of 1–10. The HRTEM image in Figure 2d gives a typical edge image of the graphene flakes in which the number of layers of graphene flakes is seven and nine, respectively. However, these graphitic layers are distorted to some extent, which reveals that some disordered structure exists in the graphene flakes. The spherical particles have a diameter range of 10–30 nm, and their content is about 30% of the products. These particles are aggregated and fused together to form a branched morphology, as shown in Figure 2e. The HRTEM image in Figure 2f shows the spherical particles possess many small and distorted graphitic layers, which are similar to the carbon black with the amorphous structure produced at a low temperature [54,55].

**Figure 3:**
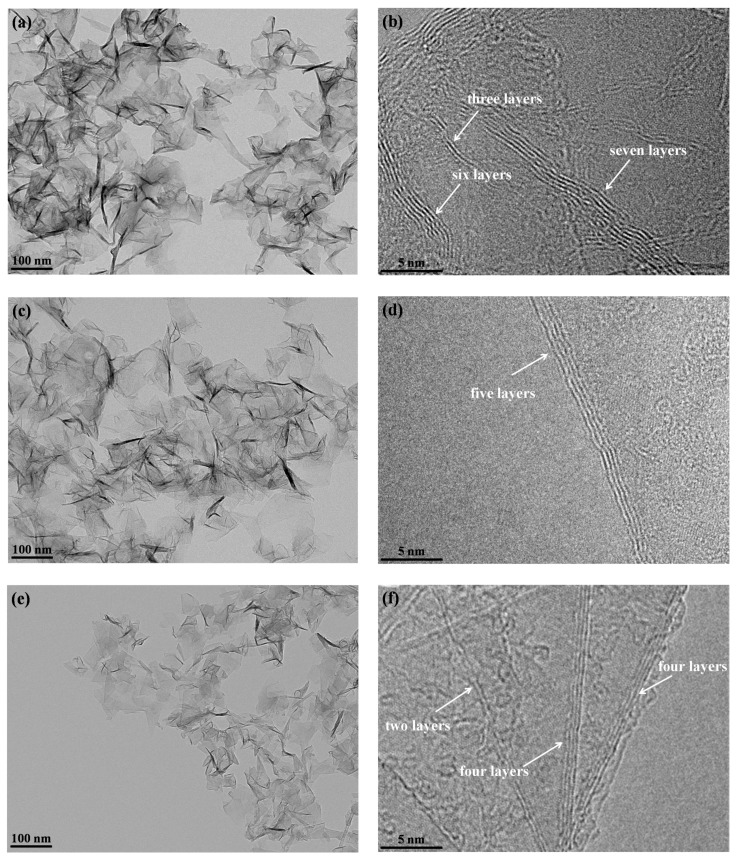
TEM images of products obtained under different buffer gases. (**a**,**b**): He, (**c**,**d**): Ar-H_2_, and (**e**,**f**): Ar-N_2_.

When the buffer gas changes, the product morphology demonstrates clear variation, as shown in Figure 3. As the buffer gas changes from Ar to He, the semi-graphitic particles and spherical particles disappear, and all products are graphene flakes ranging in size from 50 to 200 nm, as exhibited in Figure 3a. The number of layers of graphene flakes is always less than 10, as indicated by the HRTEM image in Figure 3b. Similar to the microstructure in Figure 2d, the graphene flakes should exhibit some disordered structure due to the distorted graphitic layers. When the buffer gas is Ar-H_2_, more transparent graphene flakes with a size range of 50–200 nm are obtained, as shown in Figure 3c. This phenomenon reveals that the graphene flakes have a fewer number of layers in Ar-H_2_. The HRTEM image in Figure 3d indicates the graphitic layers are straighter, which suggests a well-ordered graphitic structure in the products. When the buffer gas is Ar-N_2_, all products are graphene flakes that exhibit a smaller size (usually less than 100 nm), as shown in Figure 3e. Moreover, the graphene flakes are more transparent, which indicates fewer layers of the graphene flakes. The HRTEM image in Figure 3f reveals that the number of layers of graphene flakes is typically no more than four. In particular, the graphitic layers under Ar-N_2_ are very straight, which suggests a well-ordered graphitic structure in the graphene flakes.

### 3.2. Raman Spectroscopy

**Figure 4:**
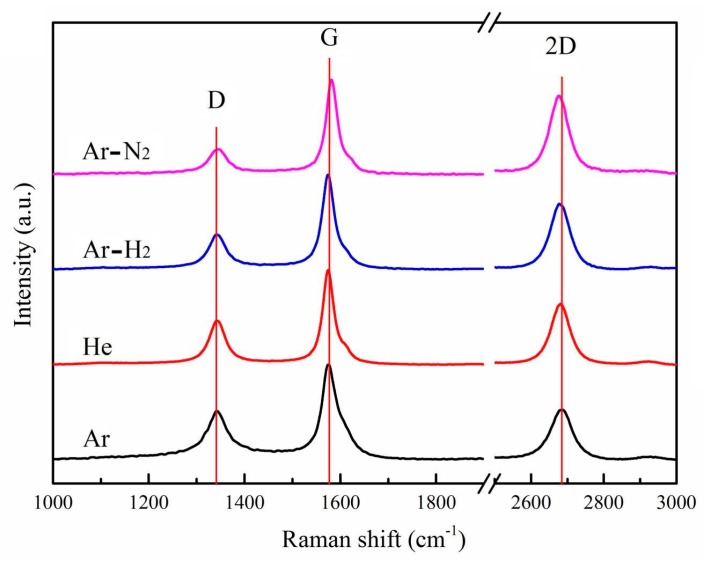
Raman spectra of products under different buffer gases.

Raman spectrum analysis of products under different buffer gases is displayed in Figure 4. As each spectrum shows, three intense peaks include the *D* band at about 1350 cm^−1^, the *G* band at about 1580 cm^−1^, and the *2D* band at about 2700 cm^−1^, respectively. The presence of the *D* band corresponds to a disordered or defective structure in carbon materials. The G band is related to phonon vibrations in sp^2^ carbon materials, which reflects the ordered graphitic sheet [56]. The *2D* band is from the overtone of the *D* band, and it is closely related to the band structure of graphene layers [57,58]. The relative intensity of the *D* band to the *G* band (*I_D_*/*I_G_*), the peak position, and the full-width at half-maximum (FWHM) of the *G* band are widely used for characterizing the defect quantity in the samples [59,60]. The relative intensity of the *2D* band to the *G* band (*I_2D_*/*I_G_*) is used to determine the thickness of graphene flakes, whereas a high value of *I_2D_*/*I_G_* indicates fewer graphene layers [5]. Table 2 summarizes the peak position and FWHM of the *G* band, the I*_D_*/I*_G_* value, and the *I_2D_*/*I_G_* value. Table 2 indicates that the peak position and FWHM of the *G* band as well as the values of I*_D_*/I*_G_* and I*_2D_*/I*_G_* are sensitive to the buffer gas. The I*_D_*/I*_G_* value under an Ar atmosphere is 0.54 and the FWHM of the *G* band is 42.37 cm^−1^, which is higher than the others. This can be attributed to the existence of spherical particles in the products. These spherical particles have an amorphous structure, which carries a high degree of defects and disorder. This results in low product crystallinity. The I*_D_*/I*_G_* value is 0.46 for He, 0.37 for Ar-H_2_, and 0.25 for Ar-N_2_, which implies increasingly high product crystallinity as the buffer gas changes. Meanwhile, the FWHM of the *G* band decreases slightly with the changes in buffer gases, which also confirms the increasingly high crystallinity. This phenomenon aligns with the variation tendency of TEM images in Figure 3 in which the graphitic layers become straighter as the buffer gas changes. These straighter graphitic layers possess a more ordered graphitic structure, which improves the crystallinity. The I*_2D_*/I*_G_* value increases as the buffer gas changes. As such, the number of layers of graphene flakes diminishes gradually, in accordance with the TEM results in Figure 3. For He, Ar-H_2_, and Ar-N_2_, all products are graphene flakes, but the FWHM of the *G* band is about 30 cm^−1^, which is higher than the single-layer graphene (~14 cm^−1^) or graphite (~12 cm^−1^) [61]. The relatively high FWHM indicates some defects and disorder in the graphene flakes [60]. According to the TEM results, the graphene flakes obtained in this experiment are small, which easily results in the formation of many wrinkles and edges, so the defects and disorder are likely a consequence of the distortion of the graphitic layers and edge effects of graphene flakes. Moreover, based on the FWHM of the *G* band, the crystal size (La) of graphene flakes can be estimated, which is about 20 nm. The *G* band for Ar-N_2_ presents a small blue shift with respect to those for other buffer gases. This phenomenon may be due to the incorporation of nitrogen into the graphene lattices, which results in the compressive/tensile strain in the C-C bonds [62,63].

### 3.3. XRD Patterns

**Figure 5:**
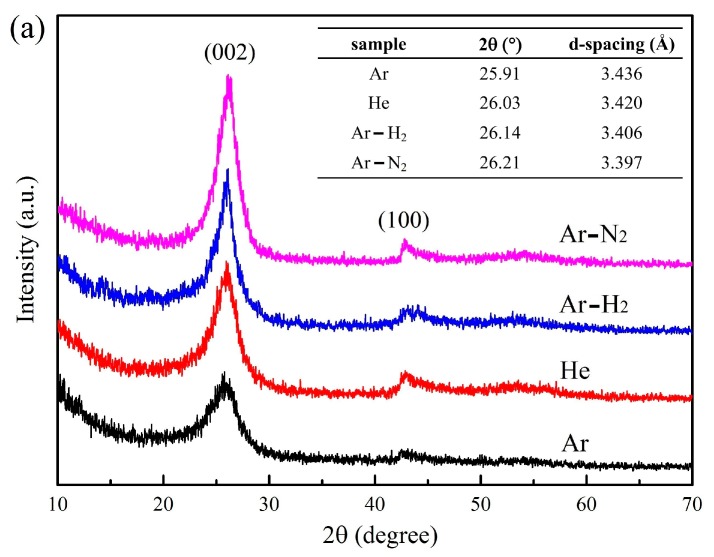

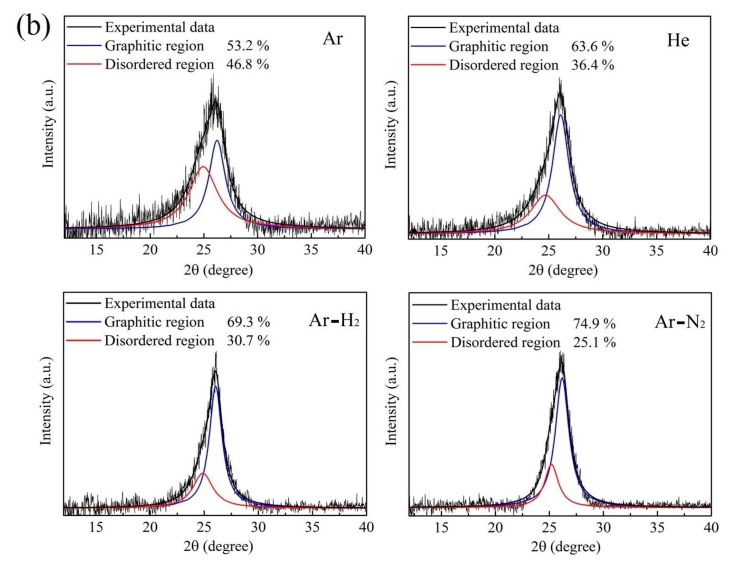
(**a**) XRD spectra. (**b**) Fitted details of d_002_ peaks of products under different buffer gases.

XRD is a useful method for the characterization of the crystal structure of nanoparticles, which is a supplement to the Raman result. XRD spectra of products obtained under different buffer gases are shown in Figure 5a. Two diffraction peaks appear at 2*θ* ≈ 26° and 2*θ* ≈ 43°, which correspond to the 002 and 100 diffraction peaks of carbon, respectively. Generally, the peak intensities of 002 and 100 are characteristic of a graphitic structure [64]. XRD spectra indicate that all products obtained in our experiment possess crystalline structures. The d-spacing, which is determined by Bragg’s equation, is also presented in Figure 5a. The products in Ar and He show 002 peaks with d-spacing of 3.436 Å and 3.420 Å at 25.91° and 26.03°, respectively. When H_2_ and N_2_ are added to the buffer gas, the products have 002 peaks with d-spacing of 3.406 Å and 3.397 Å at 26.14°and 26.21°, respectively. The shift of the 002 peak to a higher diffraction angle and declining d-spacing value further confirm a higher level of crystallinity in the products as the buffer gas changes. According to the TEM images (Figure 2 and Figure 3), the graphene flakes are composed of few graphitic layers. However, these graphitic layers are not perfect. The distorted structure always exists in the graphitic layers. Thus, the d-spacing value is usually higher than that of bulk graphite (~0.335 nm). Notably, the 002 peaks of all products are asymmetric because of a mixed polycrystalline structure of graphitic and disordered domains [65]. In order to identify the different crystal regions in the products, XRD spectra are analyzed using JADE^®^ software (version 6.5, MATERIALS DATA, California, USA) to distinguish different regions in the 002 peak. XRD analysis in Figure 5b shows two peaks in the 002 peak. One is the peak at about 2*θ* ≈ 26°, which reflects a graphitic structure, and the other is on the left, which corresponds to the disordered structure. The area from the fitting peak can describe the relative content of graphitic and disordered structures [65]. Figure 5b shows the proportions of the graphitic region under Ar and He to be 53.2% and 63.6%, respectively. For Ar-H_2_, the graphitic structure content is 69.3%, and increases to about 75% with the addition of N_2_. Combined with the morphology in TEM images, an increase in the graphitic region is mainly due to the transformation of products from spherical particles to graphene flakes, or due to the straighter graphitic layers as the buffer gas changes.

### 3.4. XPS Spectra

**Figure 6:**
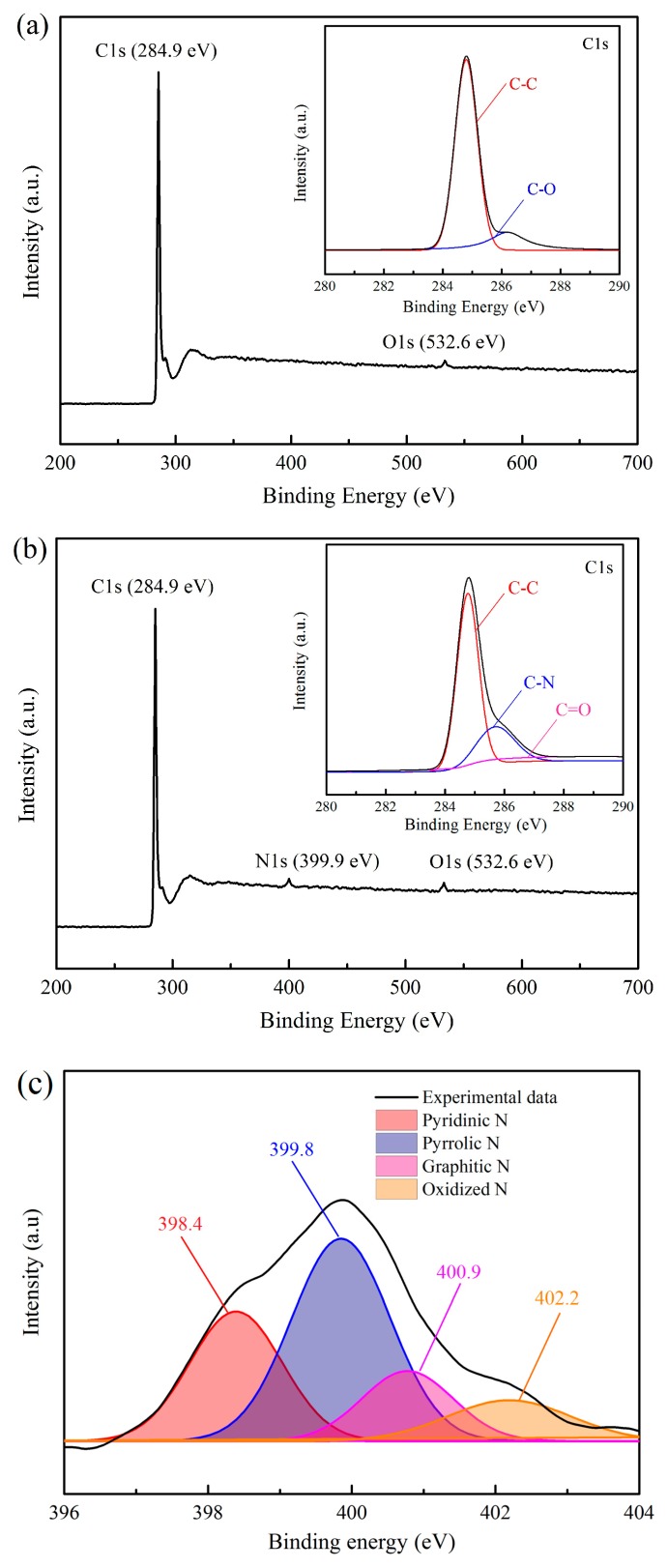
(**a**) XPS spectra of products in Ar. (**b**) XPS spectra of products in Ar-N_2_. (**c**) Fitted details of N1s peak with nitrogen addition.

The XPS spectrum can effectively reflect the elemental components of the sample surface. In our experiment, the products are prepared by an in situ synthetic method. Thus, the elemental components of the internal region and surfaces are basically the same, in principle. In this section, XPS spectra are employed to analyze the element composition of products under different buffer gases, as shown in Figure 6. Figure 6a indicates that there are two characteristic peaks: a C1s (carbon) peak at 284.9 eV and an O1s (oxygen) peak at 532.6 eV when the buffer gas is Ar. The high-resolution C1s XPS spectrum in Figure 6a can be deconvolved into two peaks centered at 284.7 and 286.4 eV, respectively. The main peak at 284.7 eV is assigned to the C-C bonds, while the minor peak at higher binding energy (286.4 eV) is very close to C-O bonds [66]. The low-intensity O1s peak and C-O bonds show a very high carbon content in the products. The XPS spectra for He and Ar-H_2_ are basically the same, and are not displayed in this case. When N_2_ is added, a small N1s (nitrogen) peak at 399.9 eV is also presented besides the C1s peak and O1s peak, as shown in Figure 6b. The high-resolution C1s XPS spectrum in Figure 6b reveals three peaks located at 284.7, 285.8, and 288.6 eV, which consists of C-C, C-N, and O-C-O/C=O groups, respectively [19,67]. Thus, the XPS spectrum indicates the formation of nitrogen-doped graphene flakes. The elemental components of products obtained in different buffer gases is shown in Table 3. For Ar, He, and Ar-H_2_, the carbon content is more than 98.7%, and the oxygen content is less than 1.3%. In our opinion, the oxygen is from the ambient air because the products are prepared in the absence of oxygen. When the products are exposed to the ambient air, the products can be oxidized by H_2_O/O_2_ species due to the existence of active sites, so as to form C-O, C-O-C, or C=O groups. For Ar-N_2_, the total amount of nitrogen incorporated into the graphene flakes is about 1.9%. The oxygen content is up to 2.3%, which is higher than those in other buffer gases. Usually, nitrogen-doped graphene flakes have more active sites [68]. This is why the products in Ar-N_2_ own a high level of oxygen content. However, the oxygen content measured by the XPS method is likely a little higher than true values. This is because the oxidation mainly occurs at the surface and edge of the products instead of the internal region. In order to distinguish the nitrogen-doped types, high resolution analysis of the N1s peak is depicted in Figure 6c. The deconvolution of this peak shows four types of N-bonding: pyridinic *N* (peak at 398.4 eV), pyrrolic *N* (peak at 399.8 eV), graphitic *N* (peak at 400.9 e V), and oxidized N (peak at 402.2 eV). Their content is 0.43%, 0.85%, 0.36%, and 0.26%, respectively.

Nitrogen-doped contents for different N_2_ flow rates are summarized in Table 4 in which the buffer gas is Ar-N_2_, and the total buffer gas flow is controlled to be 35 slm. Table 4 shows that the nitrogen-doped content rises with an increase in the N_2_ flow rate. The nitrogen-doped content is only 0.7% for 1 slm of N_2_, and rises to 1.9% for 3 slm of N_2_ and to 2.8% for 5 slm of N_2_. Noticeably, the pyrrolic *N* dominates the N-bonding types, which is more than 40% of total nitrogen-doped contents. The pyrrolic N is considered to be easily formed in a rich H environment [69], so the presence of large quantities of pyrrolic *N* is due to the abundant H atoms, which come from propane decomposition. Moreover, the pyrrolic *N* and pyridinic *N* are mainly located at the edges of graphene [70,71]. The presence of N-bonding may restrain the lateral growth of graphene by inhibiting the formation of carbon-carbon bonds at the edge. Thereby, the graphene flakes have a smaller size when the nitrogen is incorporated into the products, as shown in Figure 3.

### 3.5. BET Surface Area

**Figure 7:**
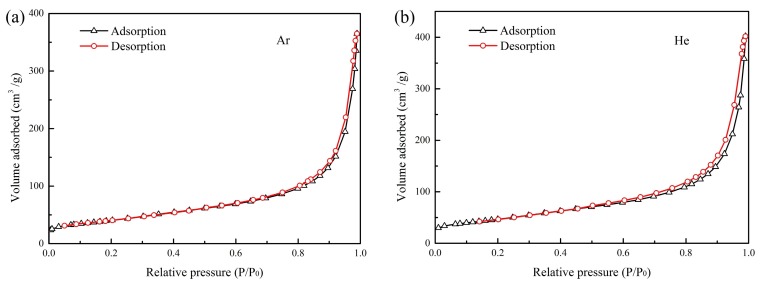

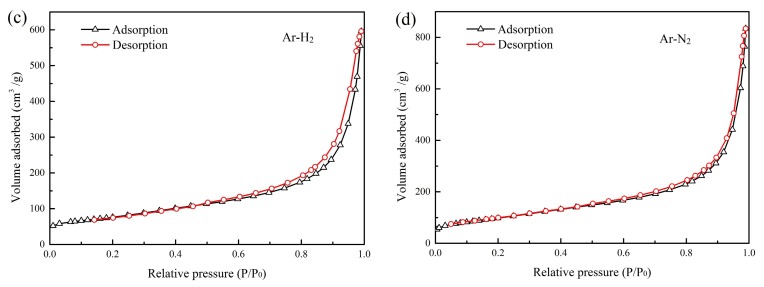
N_2_ adsorption–desorption isotherms and BET surface areas of the products obtained in different buffer gases. (**a**) Ar, (**b**) He, (**c**) Ar-H_2_, and (**d**) Ar-N_2_.

The N_2_ adsorption–desorption isotherms of the products prepared under different buffer gases are revealed in Figure 7, which indicates all the products exhibit a type IV adsorption isotherm based on the classification of International Union of Pure and Applied Chemistry (IUPAC). Each adsorption isotherm has a steep adsorption in the high relative pressure region (>0.9 P/P_0_), and presents a typical hysteresis loop in the desorption branch. The characteristic of N_2_ adsorption–desorption isotherms indicates the formation of mesopores in the products [34,72]. Considering the planar structure of graphene flakes, the predominant nitrogen adsorption at *P*/*P*_0_ > 0.9 may occur on its external surface and the internal surface of pores is formed by the re-stacking of graphene flakes. Textural data of products obtained in different buffer gases is shown in Table 5. It can be seen that the BET surface area of products obtained in Ar, He, Ar-H_2_, and Ar-N_2_ is 138.26 m^2^/g, 172.63 m^2^/g, 281.94 m^2^/g, and 353.77 m^2^/g, respectively. All products have an average pore size in the range of 13–20 nm. The products in Ar have the smallest BET surface area, which is mainly due to the presence of spherical particles and semi-graphitic particles. Usually, the specific surface area of graphene increases as the layer number decreases. As suggested by TEM images and Raman results, the layer number of graphene flakes in Ar-N_2_ is fewer than those in He and Ar-H_2_. Thus, the BET surface area of graphene flakes in Ar-N_2_ is the largest. In addition, the graphene flakes in Ar-N_2_ have the smallest average pore size. The possible reason is that the smaller graphene flakes in Ar-N_2_ are easier to form smaller mesopores compared with those produced in He and Ar-H_2_.

The BET surface area of graphene flakes prepared in this paper is lower than the theoretical value. For example, the BET surface area of graphene flakes in Ar-N_2_ is 353.77 m^2^/g, which is lower than the theoretical surface area for the four-layer graphene flakes (about 660 m^2^/g). The significant loss of accessible surface area is likely a consequence of the inhomogeneity as well as overlap or severe aggregation of graphene flakes [72]. However, the BET surface area of graphene flakes obtained in this experiment is much larger than that in the arc method in which the BET surface area is about 20–90 m^2^/g [34]. This phenomenon indicates the graphene flakes prepared in the thermal plasma process, which may possess fewer layers, less overlap, or weaker aggregation than the arc method. Therefore, the characteristic of a large specific surface area makes these graphene flakes have a good application prospect, such as catalyst carriers and super capacitors.

### 3.6. Synthesis Rate/Yield

**Figure 8:**
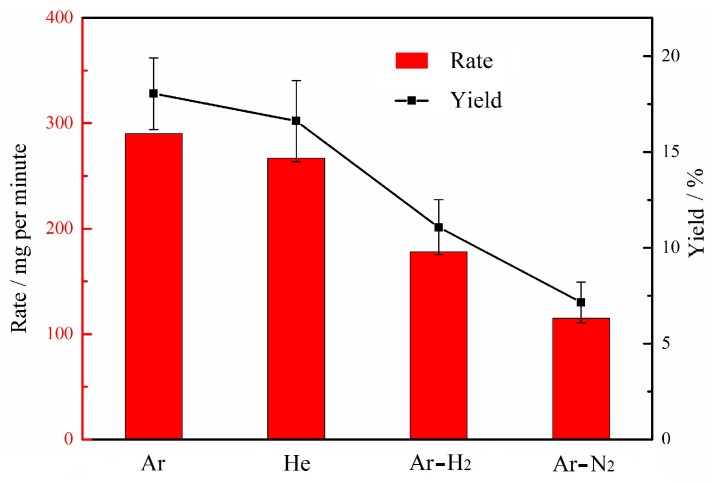
Synthesis rate and yield of carbon nanomaterials under different buffer gases.

The synthesis rate/yield of the target product is an important indicator to evaluate the thermal plasma process. Figure 8 displays that the synthesis rate/yield of carbon nanomaterials is remarkably distinct in different buffer gases. The synthesis rate is about 250–300 mg per minute in pure Ar or He atmosphere. When H_2_ is added, the synthesis rate is less than 200 mg per minute, and only about 100 mg per minute with the addition of N_2_. Accordingly, the yield of carbon nanomaterials decreases from 18% to 7%. This result indicates H_2_ and N_2_ may be involved in forming more gaseous products.

Recent research suggests that the C_2_ radicals are the main precursor species of graphene flakes [21,25,28]. In the Ar-H_2_ atmosphere, the formed C_2_ radicals can be consumed due to the formation of hydrocarbons by collision with the hydrogen molecules through the reaction [73,74]: C_2_ + H_2_ → C_2_H + H, so the synthesis rate of graphene flakes decreases. Similarly, the presence of N_2_ in plasma favors the formation of cyanides due to the interaction of nitrogen molecules with C_2_ radicals through the reaction [75,76]: C_2_ + N_2_ → CN + CN. The formation of CN species suppresses the production of C_2_ radicals. Thus, the synthesis rate of graphene flakes is also reduced. Even so, the synthesis rate/yield of graphene flakes in this paper can still compete with that in the microwave plasma process [17,21] in which the synthesis rate is about 1.33–2 mg per minute and the yield is less than 5%.

## 4. Discussion

Formation of carbon nanomaterials in the thermal plasma process is a complicated activity because the plasma environment is a complex, multi-component system including electrons, ions, atoms, molecules, and more. The formation mechanism remains unclear although some meaningful work has been made in recent years [15,20,32,52,54]. However, on the basis of product characterization by TEM, the product formation mechanism can be analyzed briefly. The spherical particles include many small and distorted graphitic layers, which implies a formation mechanism at a low temperature, similarly to the typical carbon black process [54]. Semi-graphitic particles possess graphitized forms of carbon blacks [47]. Thus, such particles are suggested to undergo a two-step process in the thermal plasma [54]: (i) primary growth of the particles at low temperature to form spherical particles, and (ii) particle graphitization in high temperature regions to cause a polyhedral morphology and shell-like appearance. The graphene flakes are mainly composed of ordered graphitic layers, corresponding to a high formation temperature [52,54]. The distorted structure in the graphitic layers emerges through the formation of five-member rings. The extent of distortion in this case is thought to be governed by competition between the formation and destruction of five-member rings, which is controlled by the plasma energy [15,77]. In thermal plasmas, the uneven distribution of temperature unavoidably exists due to the plasma fluctuation. Under an Ar atmosphere, the input power is relatively low. The uneven distribution of temperature easily causes the occurrence of a low temperature region, which leads to the formation of spherical particles. It should be noted that the semi-graphitic particles have a characteristic of near-spherical morphology, and, essentially, they belong to spherical particles with many curved or closed structures. Hence, the co-existence of three kinds of products (spherical particles, semi-graphitic particles, and graphene flakes) under an Ar atmosphere is potentially due to the uneven distribution of temperature.

As listed in Table 1, the input power for He, Ar-H_2_, and Ar-N_2_ is clearly higher than that for Ar because of the higher enthalpy for He, H_2_, and N_2_, even though the average gas temperature is always controlled to be 3500 K. Hence, the addition of high-enthalpy gas indicates an environment of high energy. In a recent study, Whitesides et al. [77] investigated the growth of graphene-edges using kinetic Monte Carlo simulations and indicated that a high-energy environment can suppress the formation of a five-member-ring, which corresponds to the curved or closed structures, and, consequently, lead to the formation of planar graphene flakes. Recent experimental studies also confirm that a high-energy environment can facilitate the formation of planar graphene flakes rather than spherical particles [11,23,27,31]. Thus, variations in gas enthalpy may represent an important factor behind changes in product morphology.

The thinner and straighter graphene flakes with the addition of H_2_ and N_2_ indicate that H_2_ and N_2_ also play key roles in graphene flakes synthesis. Several experimental studies have confirmed that H atoms are beneficial to graphene formation [38,39,78]. It is believed that H atoms can effectively terminate the dangling carbon bonds by forming carbon-hydrogen bonds, and, thus, prevent the rolling and closing of graphitic layers. In addition, H atoms have other functions of etching the amorphous carbon [79]. Thus, the formation of a spherical structure and distorted graphitic layers can be suppressed effectively in a rich H environment. In thermal plasmas, H_2_ can be easily cracked to H atoms, and the existence of H atoms has been reported in relevant research [25,29]. As a result, the addition of H_2_ facilitates the formation of straighter graphene flakes. Moreover, the thickness of the graphene flakes depends on the cooling rate in the formation process, and, normally, the faster cooling rate leads to thinner graphene flakes [37]. H_2_ has a more efficient quenching capability because its thermal conductivity is larger than that of Ar and He [80]. This may explain why the graphene flakes have fewer layers with the addition of H_2_.

The formation of nitrogen-doped graphene flakes shows an interesting result. In the arc-discharge method, the addition of N_2_ usually results in the formation of products with low crystallinity, such as spherical particles [34] and carbon nano-horns [39,81], because the carbon-nitrogen bond easily leads to the bending of graphitic layers [82,83]. On the contrary, TEM images, Raman, and XRD spectra in this experiment indicate that the graphene flakes possess greater crystallinity when applying N_2_. The variation of the synthesis rate/yield in Figure 8 offers a clue to understand this phenomenon. The addition of N_2_ may increase the relative content of H atoms since many C_2_ radicals are removed via CN species formation. The existence of large quantities of pyrrolic N in graphene flakes also indicates a rich H environment. Given this, formation of thin and straight graphene flakes in the Ar-N_2_ atmosphere is likely attributed to three factors. First, high enthalpy N_2_ can maintain a high-energy environment. Second, abundant H atoms are produced in the plasma region through propane decomposition. Third, the addition of N_2_ may improve the relative content of H atoms due to the CN species formation. Thus, the product morphology with N_2_ addition is very similar to nitrogen-doped graphene flakes produced by the arc-discharge method under an atmosphere that contains NH_3_ [35]. Nitrogen-doped graphene flakes have been found to have broad applications [70] such as an electrocatalyst for the fuel cell, a field-effect transistor, lithium ion batteries, and devices in other fields. This paper indicates that the thermal plasma process has great potential for the synthesis of nitrogen-doped graphene flakes due to its continuous manner, cheap raw materials, and adjustable nitrogen-doped content.

## 5. Conclusions

In this paper, a magnetically rotating arc plasma system is used to prepare carbon nanomaterials by propane decomposition. The products obtained in different buffer gases (i.e., Ar, He, Ar-H_2_, and Ar-N_2_) are characterized by TEM, HRTEM, Raman spectra, XRD, XPS, and the BET method. Experimental results indicate that the product microstructure depends on buffer gases. Under an Ar atmosphere, three kinds of products (spherical particles, semi-graphitic particles, and graphene flakes) coexist. Under a He atmosphere, all products transform into graphene flakes. For Ar-H_2_ and Ar-N_2_, graphene flakes with fewer layers, higher crystallinity, and larger BET surface area are obtained. As such, the buffer gas with high enthalpy, and the addition of some reactive molecules (i.e., H_2_ and N_2_), can promote the graphene flakes formation. Initial analysis indicates that a high-energy environment and abundant H atoms can prevent the formation of curved or closed structures, which produces graphene flakes with high crystallinity. In particular, nitrogen-doped graphene flakes with 1-4 layers and adjustable nitrogen-doped contents are successfully synthesized with the addition of N_2_. In summary, this study reveals that the thermal plasma process has great potential for graphene flakes synthesis because the morphology and composition of products can be effectively regulated via changes in buffer gases.

## Figures and Tables

**Table 1:** Experimental condition for each test.

Test	Input Power I/U/P	Feedstock Gas	Buffer Gas
Ar	85 A/61 V/~5.2 kW	C_3_H_8_ 1 slm	Ar 35 slm
He	92 A/103 V/~9.5 kW	C_3_H_8_ 1 slm	He 35 slm
Ar-H2	90 A/84 V/~7.6 kW	C_3_H_8_ 1 slm	Ar (32 slm), H_2_ (3 slm)
Ar-N2	95 A/78 V/~7.4 kW	C_3_H_8_ 1 slm	Ar (32 slm), N_2_ (3 slm)

**Table 2:** Raman information of products obtained in different buffer gases.

Sample	Position (cm^−1^)	FWHM (cm^−1^)	*I_D_*/*I_G_* Value	*I_2D_*/*I_G_* Value
Ar	1575.58	42.37	0.54	0.53
He	1573.28	31.40	0.46	0.64
Ar-H2	1571.17	30.81	0.37	0.69
Ar-N2	1581.56	29.36	0.25	0.82

**Table 3:** Elemental components of products obtained in different buffer gases.

Sample	C	O	N
Ar	98.9%	1.1%	-
He	98.7%	1.3%	-
Ar-H_2_	98.9%	1.1%	-
Ar-N_2_	95.8%	2.3%	1.9%

**Table 4:** Nitrogen-doped content for different N_2_ flow rates.

N_2_ Flow Rate	N-Doped Content	Pyridinic *N*	Pyrrolic *N*	Graphitic *N*	Oxidized *N*
1 slm	0.7%	0.16%	0.31%	0.12%	0.11%
3 slm	1.9%	0.43%	0.85%	0.36%	0.26%
5 slm	2.8%	0.76%	1.31%	0.41%	0.32%

**Table 5:** Textural data of products obtained in different buffer gases.

Sample	BET Surface Area (m^2^/g)	Pore Volume (cm^3^/g)	Average Pore Size (nm)
Ar	138.26	0.57	19.18
He	172.63	0.68	16.26
Ar-H_2_	281.94	0.94	14.89
Ar-N_2_	353.77	1.31	13.21
